# Supplementary material for: Deep learning of a bacterial and archaeal universal language of life enables transfer learning and illuminates microbial dark matter
Source: Nat Commun. 2022 May 11;13:2606. doi: 10.1038/s41467-022-30070-8 (PMC9095714; doi:10.1038/s41467-022-30070-8)
Supplement: Supplementary file 1 — Supplementary Information [file 41467_2022_30070_MOESM1_ESM.pdf]

Supplementary Information for the manuscript entitled:

Deep Learning of a Bacterial and Archaeal Universal Language of Life Enables Transfer Learning and Illuminates Microbial Dark Matter

Hoarfrost, A., Aptekmann, A., Farfañuk, G., Bromberg, Y.

Table of Contents for Supporting Online Material

Supplemental Figures 1-7 .... **p.1-5**

Supplemental Tables 1-6 .... **p.6-11**

Supplementary References **p.11**

Supplemental Figures

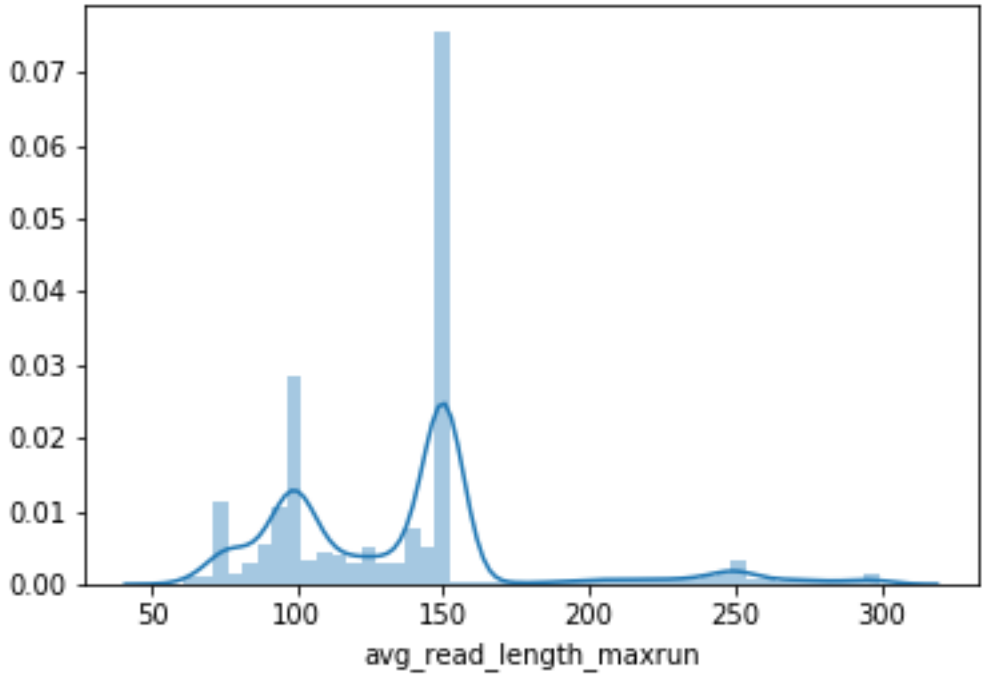

Supplementary Figure 1 – The average read length distribution of the sequencing data of the 7,909 genomes with available metadata in the Genome Taxonomy Database (GTDB)[1].

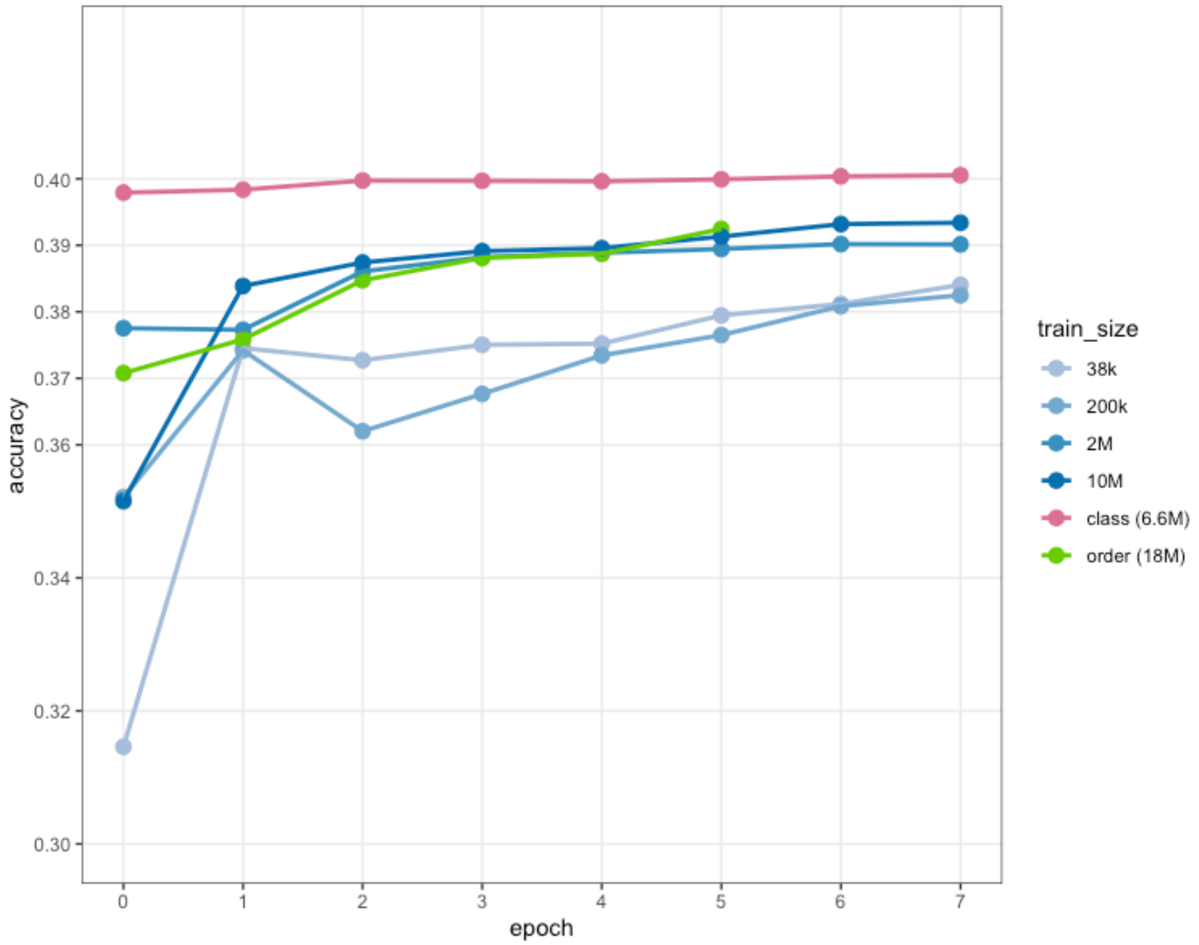

Supplementary Figure 2 – Model performance over successive epochs for progressively larger datasizes randomly subsampled from the full representative GTDB genome set (blue lines), and for all reads sourced from randomly selected genomes evenly distributed across the GTDB taxonomic tree at the order level (green) and the class level (pink). Class-level partitioning of data enables much faster convergence on maximum performance.

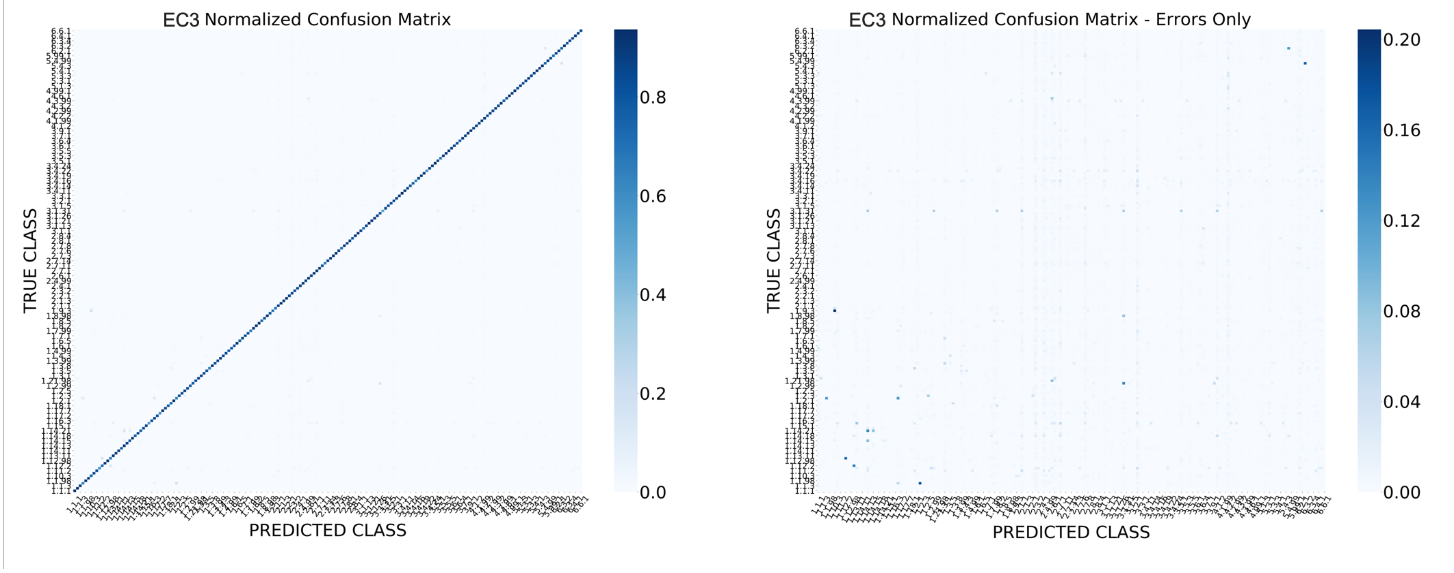

Supplementary Figure 3 - Confusion between true (y axis) and predicted (x axis) functional annotations for the functional classifier, shown as normalized percentages of predictions for each label including correct predictions (left) and showing errors only (right), for predictions to the 3rd EC number.

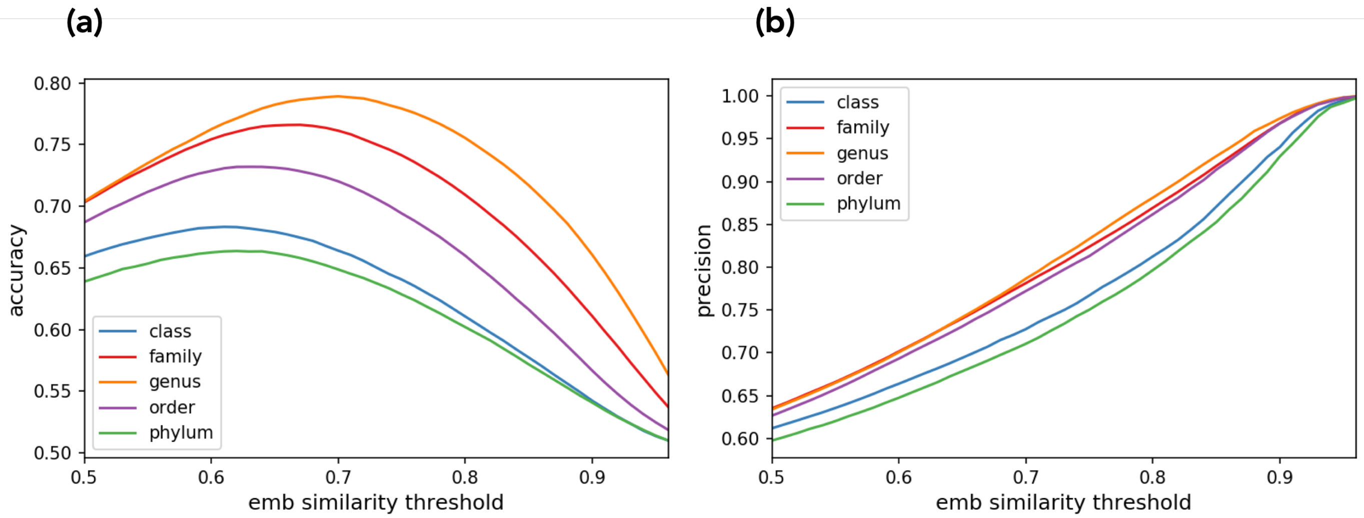

Supplementary Figure 4 – (a) Accuracy and (b) precision of predictions of whether two sequences are ‘homologous’ or ‘nonhomologous’, relative to the embedding cosine similarity threshold chosen, for each of the considered levels of taxonomic specificity.

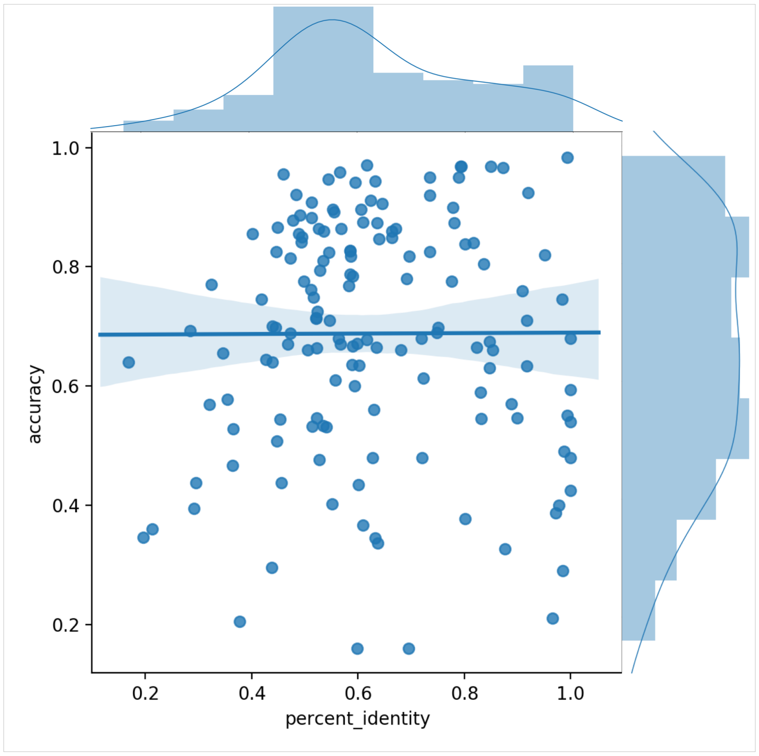

Supplementary Figure 5 – The average DNA percent identity (x axis) and oxidoreductase classifier model accuracy (y axis) for genes within each of the EC annotations in the *oxidoreductase model* validation set. Each dot represents a unique EC number.

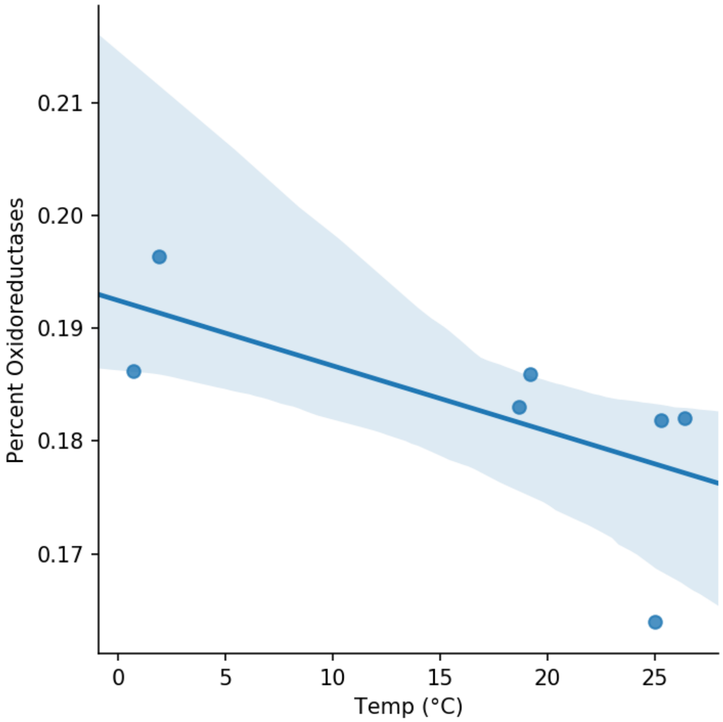

Supplementary Figure 6 – The proportion of reads identified as oxidoreductases by the oxidoreductase classifier (y axis), correlated with temperature (x axis), in surface water metagenomes from the *oxidoreductase metagenome set*. R^2 = -0.66, P=0.11.

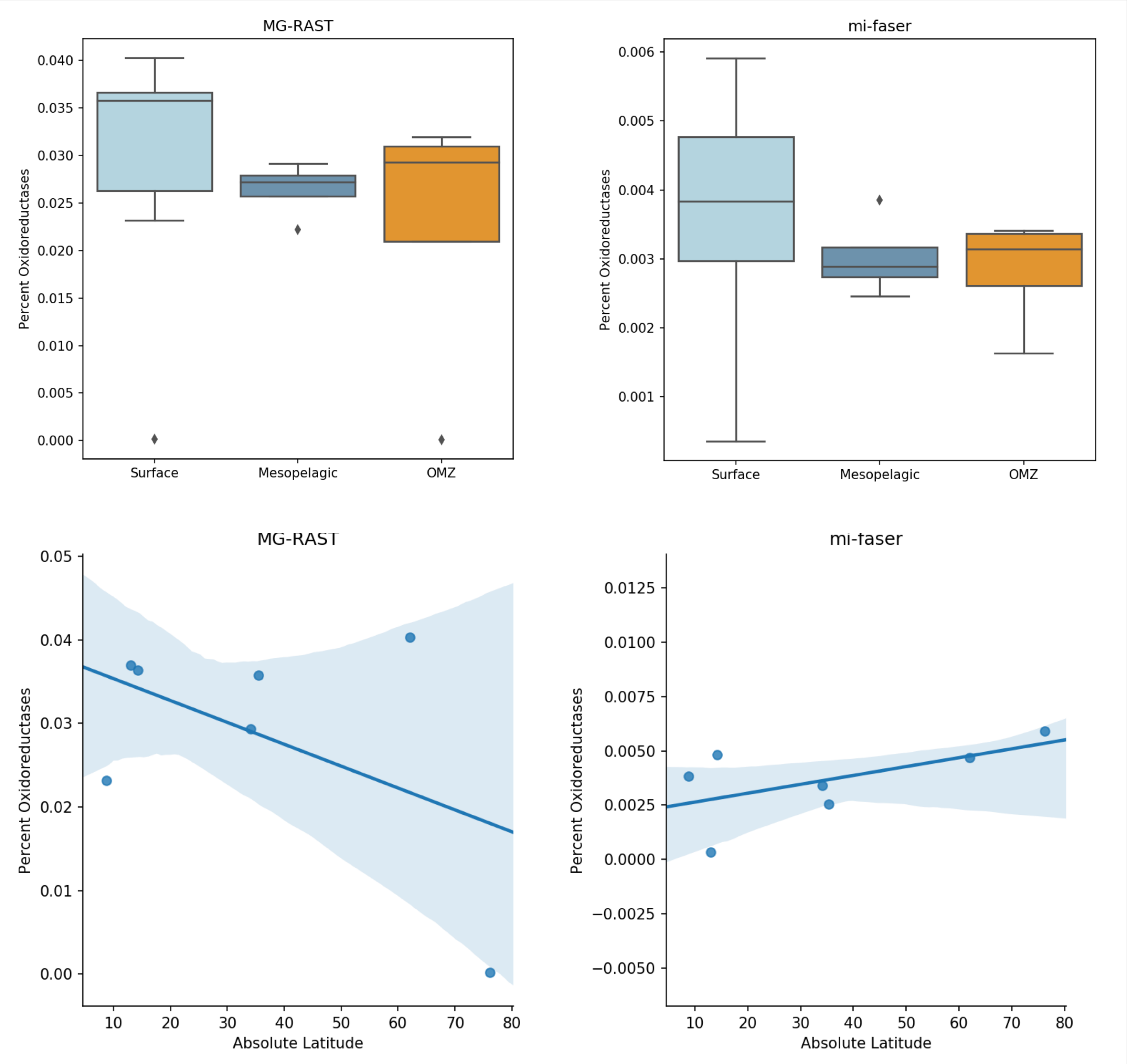

Supplementary Figure 7 – Trends in the proportion of oxidoreductases predicted in metagenomes from the *oxidoreductase metagenome set* for functional annotations from MG-RAST (left column) and mi-faser (right column). There are no significant differences in the proportion of oxidoreductases with depth (top row, ANOVA P=0.73 for MG-RAST, P=0.60 for mi-faser), and no significant trends in surface waters with latitude (bottom row, R^2= -0.49 P=0.27 for MG-RAST, and R^2=0.58 P=0.17 for mi-faser).

Supplemental Tables

Model	bs	bptt	wd	moms	Dropout rates					optim	loss fn
					input	embed	hidden	weight	output		
LookingGlass	512	100	1e-2	(0.98,0.9)	0.04	0.005	0.03	0.05	0.04	Adam ß=(0.9,0.99)	Cross Entropy
Functional annotation classifier	512	-	1e-2	(0.8,0.7)	0.12	0.015	0.09	0.15	0.12	Adam ß=(0.9,0.99)	Cross Entropy
Oxidoreductase classifier	256	-	1e-2	(0.8,.07)	0.12	0.015	0.09	0.15	0.12	Adam ß=(0.9,0.99)	Cross Entropy
Translation frame classifier	256	-	1e-2	(0.8,0.7)	0.12	0.015	0.09	0.15	0.12	Adam ß=(0.9,0.99)	Cross Entropy
Optimal temperature classifier	256	-	1e-2	(0.8,0.7)	0.12	0.015	0.09	0.15	0.12	Adam ß=(0.9,0.99)	Cross Entropy

Supplementary Table 1 – Hyperparameter settings for the LookingGlass language model and the fine tuned transfer learning classifiers. Column name abbreviations stand for: bs=batch size; bptt=back propagation through time; wd=weight decay; moms=momentums; the variable dropout rates determine the frequency of dropout applied to the dropout mask in each section of the model: input=frequency of dropout in the dropout mask for the data input matrix; embed=dropout frequency for the embedding matrix; hidden=dropout frequency for the hidden layers; weight=dropout frequency for the hidden layer weights; output=dropout frequency for the encoder output layer.

Environmental package	SRA Run Accession
built environment	ERR1332600
built environment	SRR1577782
built environment	ERR1332623
built environment	ERR1332614
built environment	ERR1332606
built environment	ERR1332586
built environment	SRR1577774
built environment	ERR2699805
built environment	ERR2699809
host-associated	SRR3184383
host-associated	SRR925829
host-associated	ERR1366724
host-associated	ERR1135232
host-associated	ERR2241851
host-associated	ERR2200504
host-associated	ERR2200674
host-associated	ERR2765138
human-gut	ERR209626
human-gut	SRR5091465
human-gut	ERR1600429
human-gut	ERR209703
human-gut	SRR5164026
human-gut	ERR1190830
human-gut	ERR3053395
human-gut	ERR2816294
human-gut	ERR3521978
microbial mat/biofilm	SRR1171647
microbial mat/biofilm	SRR1707409
microbial mat/biofilm	ERR1855538
microbial mat/biofilm	ERR1855552
microbial mat/biofilm	SRR2556884
microbial mat/biofilm	ERR1855546
microbial mat/biofilm	ERR1739691

microbial mat/biofilm	SRR830624
microbial mat/biofilm	ERR2020026
microbial mat/biofilm	ERR2020015
miscellaneous	SRR1298754
miscellaneous	DRR046818
miscellaneous	DRR027592
miscellaneous	ERR1698988
miscellaneous	ERR1353140
miscellaneous	ERR2239851
miscellaneous	ERR2298558
miscellaneous	ERR1358726
miscellaneous	ERR2239841
miscellaneous	ERR2239840
plant-associated	SRR1511001
plant-associated	SRR1754159
plant-associated	ERR2145397
plant-associated	ERR2145413
plant-associated	ERR2709726
plant-associated	ERR2969987
plant-associated	ERR2819892
plant-associated	ERR2709750
plant-associated	ERR2144884
plant-associated	ERR2022395
sediment	SRR4069407
sediment	ERR1474613
sediment	ERR1560098
sediment	ERR970605
sediment	ERR1201181
sediment	ERR1743388
sediment	ERR1743304
sediment	ERR1743339
sediment	ERR2215874
sediment	ERR1743283
soil	SRR1574704
soil	ERR1017187
soil	SRR1238204
soil	ERR1700691
soil	SRR1238205
soil	SRR5234512
soil	ERR2603191
soil	ERR1877921
soil	ERR1960504
soil	ERR2767288
wastewater/sludge	ERR712383
wastewater/sludge	SRR2938315
wastewater/sludge	ERR977414
wastewater/sludge	ERR977422
wastewater/sludge	ERR1076075
wastewater/sludge	SRR1616983
wastewater/sludge	ERR1076080
wastewater/sludge	ERR1960627
wastewater/sludge	ERR1746303
wastewater/sludge	ERR3173383
water	SRR4343439
water	ERR598955
water	ERR1726775
water	ERR1726943
water	ERR1726572
water	ERR694158
water	ERR599252
water	SRR1185414
water	ERR1987930
water	ERR2017141

Supplementary Table 2 – SRA IDs and their associated environmental packages for environmental metagenomes used for the creation of the *mi-faser functional set* (Methods).

SRA read accession	Lat	Lon	Ocean region	Depth category	Depth (m)	Temp (°C)	Oxygen (μmol/kg)	OMZ?	TARA Station
ERR598981	-12.93	-96.12	Pacific	MES	175.3	13.0	0.7	Yes	100
ERR599063	-12.99	-95.99	Pacific	SRF	5.5	25.3	200.2	-	100
ERR599115	35.31	-127.74	Pacific	MES	644.6	4.9	8.6	Yes	133
ERR599052	35.41	-127.74	Pacific	SRF	5.5	19.2	224.4	-	133
ERR599020	-1.87	-84.62	Pacific	MES	376.9	10.3	1.3	Yes	110
ERR599039	-2.01	-84.59	Pacific	SRF	5.5	23.9	190.8	-	110
ERR599076	14.17	-116.66	Pacific	MES	371.0	8.9	0.6	Yes	137
ERR598989	14.20	-116.63	Pacific	SRF	5.4	26.4	195.1	-	137
ERR599048	-8.80	-17.91	Atlantic	MES	792.7	4.7	143.2	No	072
ERR599105	-8.78	-17.91	Atlantic	SRF	5.8	25.0	199.1	-	072
ERR598964	34.10	-49.78	Atlantic	MES	734.3	10.6	155.2	No	149
ERR598963	34.10	-49.89	Atlantic	SRF	5.5	18.7	220.2	-	149
ERR599125	-61.98	-49.45	Polar	MES	783.8	0.5	203.8	No	085
ERR599176	-62.03	-49.54	Polar	SRF	5.9	0.7	343.4	-	085
ERR3589593	76.12	1.36	Polar	MES	490.5	0.1	315.4	No	163
ERR3589586	76.18	1.39	Polar	SRF	5.0	1.9	363.0	-	163

Supplementary Table 3 – SRA read accessions and associated metadata for metagenomes in the *oxidoreductase metagenome set* (Methods).

Taxlevel	Max accuracy	Max accuracy cutoff	>90% precision cutoff	>95% precision cutoff	>98% precision cutoff	R^2 emb vs. seq similarity	% pairs <50 seq similarity
**Genus**	0.789	0.7	0.82	0.88	0.91	0.44	10.7%
**Family**	0.766	0.67	0.84	0.89	0.92	0.41	17.3%
**Order**	0.732	0.63	0.84	0.89	0.92	0.37	23.2%
**Class**	0.683	0.61	0.88	0.91	0.93	0.30	45.0%
**Phylum**	0.664	0.62	0.89	0.92	0.94	0.28	44.4%

Supplementary Table 4 – Metrics for differentiation between homologous and nonhomologous sequence pairs for the five levels of taxonomic specificity tested. Columns: 'Max accuracy'=maximum accuracy across all cutoffs; 'Max accuracy cutoff'=cutoff at which maximum accuracy is achieved; '>90%, 95%, 98% precision cutoff'=cutoff at which at least 90%, 95%, or 98% precision is achieved; 'R^2 emb vs. seq similarity'=correlation coefficient between embedding cosine similarity and sequence similarity bit scores for each comparison; '% pairs <50 seq similarity'=percent of homologous sequence comparisons for which the sequence similarity bit score is less than 50.

SRA read accession	Oxidoreductase classifier	MG-RAST		mi-faser	
	% oxidoreductases	% annotated	% oxido	% annotated	% oxido
ERR599105	16.40	27.90	2.32	1.61	0.38
ERR599063	18.18	40.31	3.69	0.17	0.04
ERR598989	18.21	37.61	3.63	2.41	0.48
ERR598963	18.30	28.96	2.94	1.83	0.34
ERR599039	18.46	-	-	2.34	0.46
ERR3589593	18.47	29.04	2.69	1.82	0.29
ERR599048	18.53	30.67	2.75	1.88	0.39
ERR599052	18.59	36.86	3.58	1.27	0.25
ERR599176	18.62	50.26	4.03	2.72	0.47
ERR599020	18.74	30.13	3.07	1.94	0.33
ERR599125	19.23	26.70	2.22	1.51	0.25
ERR3589586	19.64	47.19	0.02	2.86	0.59
ERR598964	19.75	30.33	2.92	1.73	0.28
ERR599115	20.01	31.75	2.79	1.01	0.16
ERR598981	20.23	33.16	3.20	2.17	0.34
ERR599076	20.61	32.06	0.01	1.88	0.29

Supplementary Table 5 – Comparison of the % oxidoreductases predicted by the oxidoreductase classifier relative to the MG-RAST and mi-faser functional annotation tools for the metagenomes in the *oxidoreductase metagenome set,* as well as the % reads annotated overall for MG-RAST and mi-faser.

Supplementary References

1. Parks, D. H. *et al.* A standardized bacterial taxonomy based on genome phylogeny substantially revises the tree of life. *Nat. Biotechnol.* **36**, 996 (2018).
