# Supplementary material for: Deep learning of a bacterial and archaeal universal language of life enables transfer learning and illuminates microbial dark matter
Source: Nat Commun. 2022 May 11;13:2606. doi: 10.1038/s41467-022-30070-8 (PMC9095714; doi:10.1038/s41467-022-30070-8)
Supplement: Supplementary file 2 — Description of Additional Supplementary Files [file 41467_2022_30070_MOESM2_ESM.pdf]

Description of Additional Supplementary Files

File Name: Supplementary Data 1
Description: Table containing NCBI accessions for the genomes included in the *GTDB class set* (Methods).
