# Supplementary material for: Deep learning of a bacterial and archaeal universal language of life enables transfer learning and illuminates microbial dark matter
Source: Nat Commun. 2022 May 11;13:2606. doi: 10.1038/s41467-022-30070-8 (PMC9095714; doi:10.1038/s41467-022-30070-8)
Supplement: Supplementary file 4 — Reporting Summary [file 41467_2022_30070_MOESM4_ESM.pdf]

nature portfolio

Corresponding author(s): Adrienne Hoarfrost

Last updated by author(s): 02-10-2022

Reporting Summary

Nature Portfolio wishes to improve the reproducibility of the work that we publish. This form provides structure for consistency and transparency in reporting. For further information on Nature Portfolio policies, see our Editorial Policies and the Editorial Policy Checklist.

Statistics

For all statistical analyses, confirm that the following items are present in the figure legend, table legend, main text, or Methods section.

n/a	Confirmed	
☐	☒	The exact sample size (*n*) for each experimental group/condition, given as a discrete number and unit of measurement
☐	☒	A statement on whether measurements were taken from distinct samples or whether the same sample was measured repeatedly
☐	☒	The statistical test(s) used AND whether they are one- or two-sided<br>*Only common tests should be described solely by name; describe more complex techniques in the Methods section.*
☐	☒	A description of all covariates tested
☐	☒	A description of any assumptions or corrections, such as tests of normality and adjustment for multiple comparisons
☐	☒	A full description of the statistical parameters including central tendency (e.g. means) or other basic estimates (e.g. regression coefficient) AND variation (e.g. standard deviation) or associated estimates of uncertainty (e.g. confidence intervals)
☐	☒	For null hypothesis testing, the test statistic (e.g. *F*, *t*, *r*) with confidence intervals, effect sizes, degrees of freedom and *P* value noted<br>*Give P values as exact values whenever suitable.*
☒	☐	For Bayesian analysis, information on the choice of priors and Markov chain Monte Carlo settings
☐	☒	For hierarchical and complex designs, identification of the appropriate level for tests and full reporting of outcomes
☐	☒	Estimates of effect sizes (e.g. Cohen's *d*, Pearson's *r*), indicating how they were calculated

Our web collection on statistics for biologists contains articles on many of the points above.

Software and code

Policy information about availability of computer code

Data collection	all code used to collect and analyze data are open source and provided in the associated Github repository: github.com/AHoarfrost/LoL. The pretrained models are available in release v1 of: github.com/AHoarfrost/LookingGlass, and a python package (fastBio) was also developed to enable broader use of training scripts and pretrained models: github.com/AHoarfrost/fastBio. Software used: python (v3.7), mi-faser (v1.6), CD-HIT (v4.8.1), hmmscan (v2.41.2)
Data analysis	all code used to collect and analyze data are open source and provided in the associated Github repository: github.com/AHoarfrost/LoL. The pretrained models are available in release v1 of: github.com/AHoarfrost/LookingGlass, and a python package (fastBio) was also developed to enable broader use of training scripts and pretrained models: github.com/AHoarfrost/fastBio. Software used: python (v3.7), mi-faser (v1.6), CD-HIT (v4.8.1), hmmscan (v2.41.2)

For manuscripts utilizing custom algorithms or software that are central to the research but not yet described in published literature, software must be made available to editors and reviewers. We strongly encourage code deposition in a community repository (e.g. GitHub). See the Nature Portfolio guidelines for submitting code & software for further information.

Data

Policy information about availability of data

All manuscripts must include a data availability statement. This statement should provide the following information, where applicable:

- Accession codes, unique identifiers, or web links for publicly available datasets
- A description of any restrictions on data availability
- For clinical datasets or third party data, please ensure that the statement adheres to our policy

Data used for training models were produced from publicly available sources on NCBI with reference to taxonomy in GTDB and metadata in MetaSeek. Additional

datasets in Table 1 were generated from the public databases SRA, UniProt, OrthoDB, EMBL, BacDive, DSMZ, PI, and NIES. Accession codes for each sequence used in each dataset are impractical to list here but can be found in the appropriate data table in the associated github repository for this manuscript: github.com/AHoarfrost/LoL.

Field-specific reporting

Please select the one below that is the best fit for your research. If you are not sure, read the appropriate sections before making your selection.

☒ Life sciences ☐ Behavioural & social sciences ☐ Ecological, evolutionary & environmental sciences

For a reference copy of the document with all sections, see nature.com/documents/nr-reporting-summary-flat.pdf

Life sciences study design

All studies must disclose on these points even when the disclosure is negative.

Sample size	Sample sizes were determined for each dataset by how many genes/genomes were publicly available with the required parameters (described in detail in the manuscript). Where data sizes allowed, the amount of data required for adequate model performance was tuned explicitly.
Data exclusions	no data excluded
Replication	Training of models can be replicated from the scripts in the associated Github repository: github.com/ahoarfrost/LoL. Where possible, training and analyses were done with a set seed in order to ensure replicable model and analysis outputs, and these outputs were confirmed to be identical in these cases.
Randomization	training/validation/test splits were carefully chosen to test the generalization properties of trained models. How this was done for each individual dataset is described in more detail in the manuscript; in some cases, datapoints were split randomly; where appropriate, datapoints were split along taxonomy such that no taxonomic group in the validation or test sets were contained in the training set, to ensure generalizability and avoid overfitting of models.
Blinding	Blinding is not relevant to this study, since individuals are not performing the model training manually; however, blinding is simulated in the learning of the models in the careful training/validation/test splits, in which data in the validation and test sets are not seen during training.

Reporting for specific materials, systems and methods

We require information from authors about some types of materials, experimental systems and methods used in many studies. Here, indicate whether each material, system or method listed is relevant to your study. If you are not sure if a list item applies to your research, read the appropriate section before selecting a response.

Materials & experimental systems

n/a	Involved in the study	
☒	☐	Antibodies
☒	☐	Eukaryotic cell lines
☒	☐	Palaeontology and archaeology
☒	☐	Animals and other organisms
☒	☐	Human research participants
☒	☐	Clinical data
☒	☐	Dual use research of concern

Methods

n/a	Involved in the study	
☒	☐	ChIP-seq
☒	☐	Flow cytometry
☒	☐	MRI-based neuroimaging
